# Alzheimer’s and neurodegenerative disease biomarkers in blood predict brain atrophy and cognitive decline

**DOI:** 10.1186/s13195-024-01459-y

**Published:** 2024-04-30

**Authors:** Heather E. Dark, Yang An, Michael R. Duggan, Cassandra Joynes, Christos Davatzikos, Guray Erus, Alexandria Lewis, Abhay R. Moghekar, Susan M. Resnick, Keenan A. Walker

**Affiliations:** 1 Laboratory of Behavioral Neuroscience, National Institute On Aging, NIH BRC BG RM 04B311, 251 Bayview Blvd, Baltimore, MD 21224 USA; 2 Department of Radiology, University of Pennsylvania, Philadelphia, PA USA; 3 Department of Neurology, Johns Hopkins University School of Medicine, Baltimore, MD USA

**Keywords:** Plasma biomarkers, Brain atrophy, Cognitive decline

## Abstract

**Background:**

Although blood-based biomarkers have been identified as cost-effective and scalable alternatives to PET and CSF markers of neurodegenerative disease, little is known about how these biomarkers predict future brain atrophy and cognitive decline in cognitively unimpaired individuals. Using data from the Baltimore Longitudinal Study of Aging (BLSA), we examined whether plasma biomarkers of Alzheimer’s disease (AD) pathology (amyloid-β [Aβ_42/40_], phosphorylated tau [pTau-181]), astrogliosis (glial fibrillary acidic protein [GFAP]), and neuronal injury (neurofilament light chain [NfL]) were associated with longitudinal brain volume loss and cognitive decline. Additionally, we determined whether sex, *APOE*ε4 status, and plasma amyloid-β status modified these associations.

**Methods:**

Plasma biomarkers were measured using Quanterix SIMOA assays. Regional brain volumes were measured by 3T MRI, and a battery of neuropsychological tests assessed five cognitive domains. Linear mixed effects models adjusted for demographic factors, kidney function, and intracranial volume (MRI analyses) were completed to relate baseline plasma biomarkers to baseline and longitudinal brain volume and cognitive performance.

**Results:**

Brain volume analyses included 622 participants (mean age ± SD: 70.9 ± 10.2) with an average of 3.3 MRI scans over 4.7 years. Cognitive performance analyses included 674 participants (mean age ± SD: 71.2 ± 10.0) with an average of 3.9 cognitive assessments over 5.7 years. Higher baseline pTau-181 was associated with steeper declines in total gray matter volume and steeper regional declines in several medial temporal regions, whereas higher baseline GFAP was associated with greater longitudinal increases in ventricular volume. Baseline Aβ_42/40_ and NfL levels were not associated with changes in brain volume. Lower baseline Aβ_42/40_ (higher Aβ burden) was associated with a faster decline in verbal memory and visuospatial performance, whereas higher baseline GFAP was associated with a faster decline in verbal fluency. Results were generally consistent across sex and *APOE*ε4 status. However, the associations of higher pTau-181 with increasing ventricular volume and memory declines were significantly stronger among individuals with higher Aβ burden, as was the association of higher GFAP with memory decline.

**Conclusions:**

Among cognitively unimpaired older adults, plasma biomarkers of AD pathology (pTau-181) and astrogliosis (GFAP), but not neuronal injury (NfL), serve as markers of future brain atrophy and cognitive decline.

**Supplementary Information:**

The online version contains supplementary material available at 10.1186/s13195-024-01459-y.

## Introduction

Alzheimer’s disease (AD) is a debilitating neurodegenerative condition characterized by the presence of extracellular amyloid-β (Aβ) plaques, intracellular tau-containing neurofibrillary tangles (NFTs), neurodegeneration, and cognitive impairment [1, 2]. Traditionally, hallmarks of AD have been measured in vivo using positron emission tomography (PET) imaging or cerebral spinal fluid (CSF) measurement. Biomarkers of AD pathology as well as non-specific markers of neurodegeneration are now measurable in blood using ultrasensitive assays, providing a cost-effective, scalable, and minimally invasive alternative to PET and CSF measurement. Several studies demonstrate that plasma biomarkers of AD pathology (e.g., Aβ_42/40_, phosphorylated tau [pTau-181]), astrogliosis (glial fibrillary acidic protein [GFAP]), and to a lesser extent, neuronal injury (neurofilament light chain [NfL]) relate to – and can be used to predict – cortical Aβ and tau burden [3–6]. Further, plasma pTau-181, GFAP and NfL differentiate between AD, mild cognitive impairment, and control participants [7–9], and plasma pTau-181 and GFAP increase over time in preclinical AD [10]. Although plasma Aβ_42/40_ predicts Aβ-PET positivity [11, 12], its utility for differentiating clinically-defined disease stages is comparatively limited [13, 14]. These blood biomarkers are poised to play an important role in dementia research and clinical practice and are already being used to screen participants and as secondary endpoints in clinical trials for AD [15]. While these blood biomarkers have been extensively characterized in recent years, the prognostic significance of these measures among cognitively normal older adults remains largely unknown.

Prior cross-sectional studies have shown that that higher NfL [7, 16], GFAP [5, 17], pTau-181 [18], and lower Aβ_42/40_ [5] in blood were associated with smaller brain volume in regions vulnerable to AD (e.g., hippocampus, entorhinal cortex), as well as white matter volume [19]. Additionally, higher blood levels of NfL and GFAP [7, 17] and longitudinal increases blood levels of NfL [16, 20, 21] and pTau-181 [18] have been associated with decreased volume in AD-vulnerable brain regions.

Although previous studies have shown that lower Aβ_42/40_ and higher ptau-181 [18, 22], NfL [7, 17], and GFAP [17] in blood are associated with increased rate of cognitive decline [23], the vast majority of studies connecting these blood biomarkers to cognitive function have been cross-sectional [7, 17, 19, 24]. Accordingly, the extent to which these measures relate to future brain volume loss and cognitive decline in a cognitively normal community sample is not well understood. Additionally, whether demographic, genetic, and disease staging factors modify these associations is unknown. To address these questions, we used data from the Baltimore Longitudinal Study of Aging (BLSA) to examine the association of Aβ_42/40_, pTau-181, GFAP, and NfL with baseline and longitudinal measures of regional brain volume and domain-specific cognition. We further sought to determine whether sex, *APOE*ε4 carrier status, and plasma amyloid status modified the association between plasma biomarkers and longitudinal brain volume and cognition.

## Methods

### Participants

**Fig. 1:**
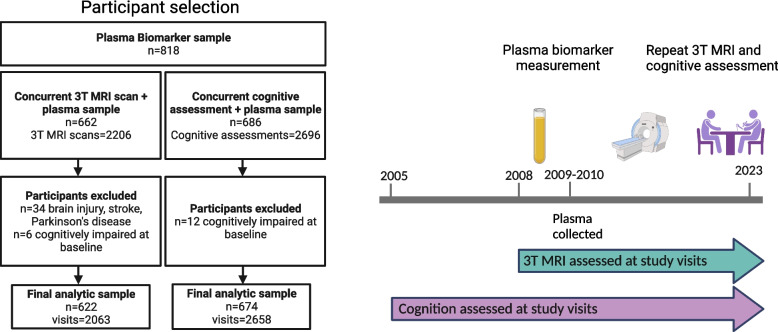
Study design and participant selection. Participants completed 3T MRI beginning in 2008, and the standardized cognitive battery beginning in 2005. The majority of plasma biomarker measurements occurred in 2009; however, some participant samples were as early as 2005. For MRI analyses, the first plasma biomarker visit for participants on or after age 50 at the time of collection, that also had a concurrent 3T MRI scan was selected as the baseline visit for brain volume analyses. Similarly, for cognition analyses, the first plasma biomarker visit for participants on or after age 50 at the time of collection, that also had a concurrent cognitive assessment was selected as the baseline visit for cognition analyses. Fig.created with BioRender.com

The current study used data from the BLSA, an ongoing community-based longitudinal study of physical and psychological aging [25]. Recruitment and enrollment for the BLSA have been previously described [26, 27]. BLSA study visits occur every four years for participants aged < 60 years, every two years for participants aged 60–79 years, and every year for participants aged ≥ 80 years. At each visit participants receive a clinical and physiological assessment, health and lifestyle questionnaires, and a comprehensive cognitive exam. BLSA participants received 3T MRI scans at each visit (beginning in 2008) as part of the Neuroimaging Substudy. Blood samples were collected at each participant’s first 3T MRI visit, and for a set of participants, during the first PET visit as a part of a separate protocol. We selected the first biomarker visit (on and after age 50 at the time of collection) that also had a concurrent 3T MRI scan as the baseline for brain volume analyses. For the cognition sample, we selected the first biomarker visit (on and after age 50 at the time of collection) that had a concurrent cognitive assessment as the baseline for the cognition analyses. Participants were included if they were cognitively unimpaired at baseline, had 3T MRI data or neuropsychological data, were at least 50 years old, and did not have neurological health conditions (e.g., stroke, seizures, previous brain surgery) or MRI contraindications (Fig. 1). Mild cognitive impairment (MCI) and dementia classification were determined by consensus diagnostic conference, as described previously [28]. Briefly, a participant’s cognitive status was adjudicated at consensus diagnostic conference if their score on the Blessed Information-Memory-Concentration Test [29] score was ≥ 4, if their self or informant Clinical Dementia Rating (CDR) scale [30] was ≥ 0.5, and/or if concerns were raised about their cognition by study staff. Impairment was determined using selected longitudinal neuropsychological assessment and CDR scores. Cognitive tests are listed elsewhere [28]. Mild cognitive impairment was diagnosed using the Petersen criteria: 1) when cognitive impairment was present for a single domain (typically memory), or 2) when cognitive impairment occurred without significant functional loss in activities of daily living [31]. Diagnoses of dementia are consistent with the Diagnostic and Statistical Manual, third edition, revised. [32]

#### MRI

 Participants in the BLSA Neuroimaging Substudy completed T1-weighted magnetization-prepared rapid gradient echo (MPRAGE) scans (TR = 6.8 ms, TE = 3.2 ms, flip angle = 8◦, image matrix = 256 × 256 × 170, voxel size = 1.0 × 1.0 × 1.2 mm^3^) on a 3 T (Philips Achieva) over an average of 4.7 years (Table 1). Preprocessing procedures have been described elsewhere [27]. Anatomical regions of interest (ROIs) were segmented using the Multi-atlas Region Segmentation Utilizing Ensembles (MUSE) anatomic labeling method to create voxelwise tissue maps reflecting local volumes (RAVENS maps) [33, 34]. MUSE ROI labels were used for the primary analysis in the current study. ROIs for the present study included total brain, gray matter, and white matter volumes, ventricular volume, superior, middle, and inferior temporal gyrus volume, as well as parahippocampal gyrus, hippocampal, entorhinal cortex, and amygdala volumes. Whole brain RAVENS gray matter maps (smoothed using a 2 mm Gaussian filter) were used for secondary voxel-based morphometry (VBM) analyses. VBM analyses were completed using AFNI 16.3.13, FSL 5.0.6, and MatLab 2018b.

**Table 1:** Participant characteristics

Baseline characteristics	MRI sample		Cognition sample	
	Full analytic sample	Sub-sample with pTau-181	Full analytic sample	Sub-sample with pTau-181
n	622	574	674	602
age	70.9 ± 10.2 (50.1–96.2)	69.7 ± 10.6 (50.1–96.2)	71.2 ± 10.0 (50.1–94.7)	70.0 ± 10.6 (50.1–96.2)
female	347 (55.8%)	318 (55.4%)	365 (54.2%)	331 (55.0%)
White	417 (67.0%)	372 (64.8%)	462 (68.6%)	395 (65.6%)
Black	167 (26.9%)	161 (28.1%)	172 (25.5%)	165 (27.4%)
Other race	38 (6.1%)	41 (7.1%)	40 (5.9%)	42 (7.0%)
Education	17.0 ± 2.4 (7–21)	17.0 ± 2.4 (7–21)	17.0 ± 2.4 (7–21)	17.0 ± 2.4 (7–21)
*APOE* genotype				
0 ε4 alleles	433 (69.6%)	397 (69.2%)	469 (69.6%)	420 (69.8%)
1 or 2 ε4 alleles	153 (24.6%)	140 (24.4%)	169 (25.0%)	145 (24.1%)
Missing	36 (5.8%)	37 (6.4%)	36 (5.3%)	37 (6.1%)
Incident MCI or dementia	39 (6.3%)	32 (5.6%)	57 (8.5)%	47 (7.8%)
eGFR	79.5 ± 15.1 (25.0–110.0)	80.6 ± 14.9 (25.0–110.0)	79.1 ± 15.0 (25.0–110.0)	80.4 ± 14.8 (25.0–110.0)
Aβ_42/40_	0.054 ± 0.012 (0.013–0.083)	-	0.054 ± 0.012 (0.013–0.083)	-
Low Aβ_42/40_/ High Aβ_42/40_	245 (39.5%)/376 (60.6%) 1 missing	196 (36.2%)/346 (63.8%) 32 missing	272 (40.4%)/401 (59.6%) 1 missing	214 (37.5%)/356 (62.5%) 32 missing
GFAP	172.0 ± 87.4 (32.6–734.8)	-	174.5 ± 85.7 (32.6–686.1)	-
NfL	22.1 ± 11.0 (4.4–76.4)	-	22.7 ± 11.4 (5.2–76.4)	-
pTau-181	-	2.57 ± 1.40 (0.36–9.83)	-	2.58 ± 1.37 (0.36–9.83)
Longitudinal characteristics				
Total observations	2063	1812	2658	2265
Follow up time (years)	4.7 ± 2.9 (0–10.8)	4.5 ± 2.9 (0–10.8)	5.7 ± 3.2 (0–16.5)	5.6 ± 3.3 (0–13.1)
Assessments per participant	3.3 ± 1.7 (1–11)	3.2 ± 1.6 (1–10)	3.9 ± 2.1 (1.0–13.0)	3.8 ± 2.0 (1.0–12.0)
Assessments per participant for those with 2 or more visits (longitudinal data)	3.7 ± 1.5 (2–11)	3.6 ± 1.4 (2–10)	4.3 ± 1.9 (2–13)	4.2 ± 1.8 (2–12)

Data are presented as mean ± standard deviation (range), or frequency (percentage)

*Abbreviations:* Aβ amyloid-β, eGFR Estimated glomerular filtration rate, GFAP Glial fibrillary acidic protein, MCI mild cognitive impairment, MRI Magnetic resonance imaging, NfL Neurofilament light chain, pTau-181 Tau phosphorylated at threonine 181

### Neuropsychological assessment

Cognitive performance was assessed using composite scores across five cognitive domains: attention, verbal fluency, visuospatial skills, verbal memory, and executive functioning. Scores from individual cognitive measures were z-scored using the baseline mean and standard deviation and averaged to create each domain composite score. The attention composite was comprised of scores from Trail Making Test Part A (total seconds)^35^ and the Digit Span Forward subtest (total score) from the Wechsler Adult Intelligence Scale-Revised [36]. The verbal fluency composite included both letter (F,A,S) and category fluency (fruits, animals, vegetables), which each consisted of total words recalled in 60-s. The visuospatial composite included a modified version of the Educational Testing Service Card Rotations Test (total score = total number correct – total number incorrect) [37]. The verbal memory composite was created using immediate recall (sum of 5 trials) and long-delay free recall from the California Verbal Learning Test-I [38]. Long delay free recall included a 20-min delay. The executive function composite included Trail Making Test Part B (total seconds)^35^ and the Digit Span Backward subtest (total score) from the Wechsler Adult Intelligence Scale-Revised [36]. Prior to computing the composites, Trails scores (in seconds) were inverted so higher scores reflect better performance.

### Plasma biomarkers

Aβ_40_, Aβ_42_, GFAP, NfL and pTau-181 concentrations were measured using the Single Molecule Array (Simoa®) Neurology 4-Plex E (N4PE) and pTau-181 (V2) assays on the Simoa HD-X instrument (Quanterix™ Corporation). Assays were run in duplicate, and the values were averaged. Intra-assay coefficients of variation (CVs) for Aβ_40_, Aβ_42_, GFAP, NfL and pTau-181 were 1.5%, 1.0%, 4.9%, 4.8%, and 4.4%, respectively, while inter-assay CVs were 5.2%, 5.9%, 8.1%, 7.8%, and 13.1%, respectively. Outliers were defined as 5 standard deviation outside the mean, and were excluded. Aβ_42/40_ status (low/high) was determined using a cutoff value (0.05259) derived from an ROC analysis to determine the optimal threshold (Youden’s Index) for predicting amyloid (^11^C-Pittsburgh compound-B [PiB]) PET positivity in a subsample of participants (n = 212) from the present study. All BLSA (Neuroimaging Substudy) participants with available PiB PET data (n = 212) were used for the analysis. Participants from the BLSA were eligible to undergo PiB PET imaging if they were not diagnosed with CNS disease, severe cardiac (myocardial infarction, coronary artery disease requiring angioplasty or coronary artery bypass surgery) or pulmonary disease, and metastatic cancer [39]. Full details of PET acquisition and preprocessing have been described elsewhere [40]. The cutoff to determine PiB status was a mean cortical distribution volume ratio (DVR) of 1.064 derived from a Gaussian mixture model. Participants with DVR > 1.064 were considered PiB positive, while those below were considered PiB negative.

### Genotyping

*APOE*ε4 carrier status was determined by either polymerase chain reaction (PCR) with restriction isotyping using the Type IIP enzyme Hhai [41] or the Taqman method [42]. Participants were classified as ε4 non-carrier (0 ε4 alleles), or carrier (1–2 ε4 alleles).

### Covariates

Baseline age, sex (male/female), race (white/non-white), and education level (total years) were defined based on participant self-report. Estimated glomerular filtration rate (eGFR)-creatinine, used as a proxy for kidney function, was defined using the Chronic Kidney Disease Epidemiology Collaboration (CKD-EPI) criteria [43, 44]. Total intracranial volume (ICV) at age 70 was computed using methods previously described [45] and included as a covariate in ROI and VBM analyses.

### Statistical analyses

Separate linear mixed effects (LME) models were used to examine the associations of baseline values of each plasma biomarker of AD pathology and neurodegeneration (predictor variables) with baseline and longitudinal change in brain volumes and cognitive performance (outcome variables). Each model adjusted for baseline age, sex, race, education, and eGFR, as well as the interaction of each covariate with time (of follow up) to adjust for longitudinal effects of confounders. Random effects included intercept and time with an unstructured covariance matrix. Analyses of brain volumes also adjusted for intracranial volume defined at age 70. Plasma biomarkers and brain volumes were standardized (mean = 0 and standard deviation = 1). Secondary analyses examined the modifying effect of sex, *APOE*ε4 status, and Aβ_42/40_ status on the association of plasma biomarkers with brain volume and cognitive trajectories by including additional two-way (modifier*biomarker) and three-way (biomarker*modifier*time) interactions in models. Modifier-specific estimates were derived within the same model using linear combinations of relevant beta coefficients. Additional sensitivity analyses were performed without eGFR as a covariate even though kidney function has been previously shown to impact plasma biomarker concentration [46–48] likely due to poor renal clearance [49]. All continuous covariates were mean centered, sex was coded 0.5 for males and -0.5 for females. Race was coded using 3 categories: White, Black and other race, where White was used as the reference group. All participants were cognitively unimpaired at baseline. A false discovery rate (FDR) method was used to correct for multiple comparisons. Statistical significance was defined as FDR-corrected *P* < 0.05 for primary analyses and as uncorrected *P* < 0.05 for secondary analyses. All statistical analyses were performed using SAS statistical software version 9.4 (SAS institute). Linear mixed effects models conducted for VBM mirrored ROI analyses.

## Results

### Baseline plasma biomarkers and brain volume

Participant characteristics are described in Table 1 (Aβ_42/40_, GFAP, and NfL sample: N = 622, mean age = 70.9 ± 10.2, 55.8% female, 67.0% White; pTau-181 sample: N = 574, mean age = 69.7 ± 10.6, 55.4% female, 64.8% White). Follow up time was 4.7 years (SD = 2.9) for Aβ_42/40_, GFAP, and NfL and 4.5 years (SD = 2.9) for pTau-181. Thirty-nine (6%) participants developed cognitive impairment during follow up. Kidney function, as estimated by eGFR, was inversely associated with pTau-181 (ρ = -0.23, *P* < 0.0001) and NfL (ρ = -0.21, *P* < 0.0001) levels, but did not correlate with Aβ_42/40_ (ρ = 0.02, *P* = 0.64) and GFAP (ρ = -0.07, *P* = 0.06).

**Fig. 2:**
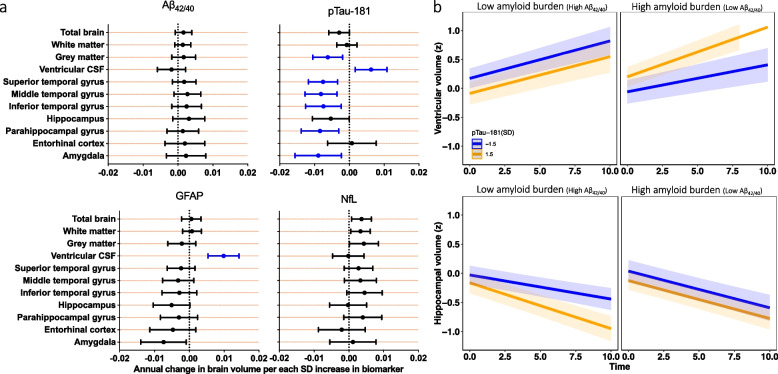
Association between baseline plasma biomarkers and longitudinal brain volume change. **a.** Results are derived from linear mixed effect models adjusted for baseline age, sex, race, education level, estimated glomerular filtration rate (eGFR), total intracranial volume at age 70, and the interaction between covariates and time. Estimates, derived from the plasma biomarker × time interaction terms, represent the difference in annual change in brain volume (β estimates) per standard deviation (SD) increase in baseline biomarker. Blue bars reflect significant associations between plasma biomarkers and change in brain volume at an FDR corrected threshold (*P* < 0.05). **b.** Results are derived from linear mixed effect models that examined Aβ_42/40_ as a moderator of the association between plasma biomarkers and longitudinal change in brain volume (models were also adjusted for, baseline age, sex, race, education level, eGFR, total intracranial volume at age 70, and the interaction between covariates and time). Each figure (blue lines: low pTau-181 or -1.5 standard deviations below the mean; orange lines: high pTau-181 or + 1.5 standard deviations above the mean) shows the marginal effects for the following interaction term pTau-181 × time × Aβ_42/40_ status on brain volume. *Abbreviations*: Aβ: amyloid-β; CSF: cerebral spinal fluid; GFAP: glial fibrillary acidic protein; NfL: neurofilament light chain; pTau-181: tau phosphorylated at threonine-181

**Fig. 3:**
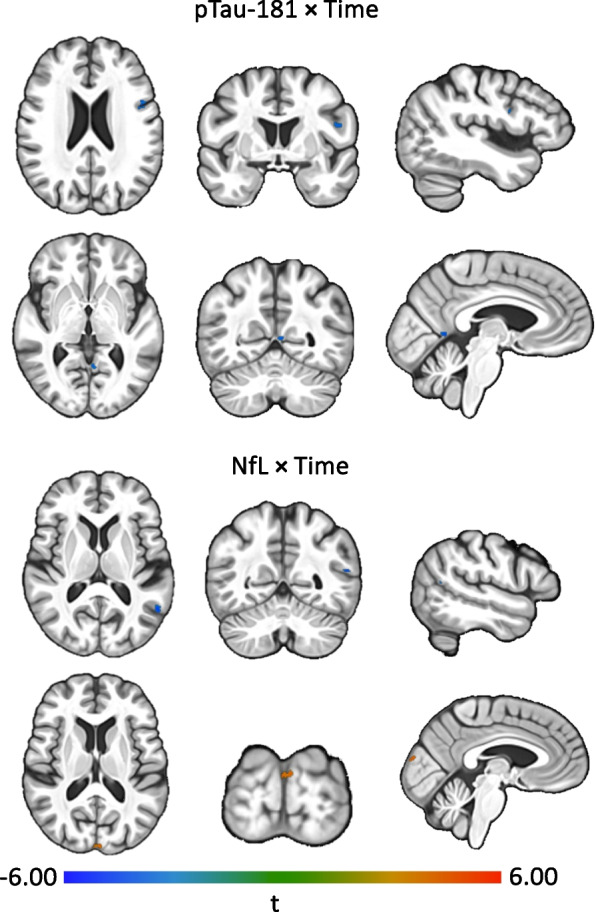
Association between plasma biomarkers and longitudinal brain volume change defined using voxel-based morphometry (VBM)**.** Plasma biomarker effects on the difference in annual change in gray matter volume (VBM). Results are derived from linear mixed effects models examining the association of baseline plasma biomarkers with longitudinal change in whole brain volume. Models were adjusted for baseline age, sex, race, education level, estimated glomerular filtration rate (eGFR), and total intracranial volume at age 70, as well as time × covariate interactions. T values reflect estimates derived from the plasma biomarker × time interactions term. Warm colors reflect positive t values while cool colors reflect negative t values. An uncorrected *P* < 0.001 threshold was used

Cross-sectional analyses showed no associations of plasma Aβ_42/40_, GFAP, and NfL with total or regional brain volumes (Supplemental Table 1; Supplemental Table 2 [model without eGFR as a covariate]; Supplemental Table 3 [full model]); however, higher plasma pTau-181 was associated with smaller inferior temporal gyrus volume (*P* < 0.0001) at baseline. pTau-181 levels were additionally associated with faster declines in total gray matter volume (*P* = 0.005), and in the superior temporal gyrus (*P* = 0.001), middle temporal gyrus (*P* = 0.001), inferior temporal gyrus (*P* = 0.005), parahippocampal gyrus (*P* = 0.002), and amygdala (*P* = 0.009) regions after correcting for multiple comparisons (Fig. 2a; Supplemental Table 1). Higher baseline GFAP was associated with faster longitudinal increases in ventricular volume (*P* < 0.0001), and a nominally significant association with steeper declines in amygdala volume which did not survive FDR correction for multiple comparisons. Aβ_42/40_ and NfL levels were not associated with brain volume change (Supplemental Table 1; Fig. 2). The results were similar in sensitivity analyses that did not adjust for eGFR (Supplementary Table 2). Secondary whole brain VBM analyses were completed to confirm the regional specificity of biomarker-associated changes in volume. As shown in Fig. 3, significant (uncorrected at *P* = 0.001) longitudinal associations with gray matter volume were observed for both pTau-181 (left inferior frontal gyrus, left precuneus) and NfL (left superior temporal gyrus, left cuneus).

Examination of effect modification by sex found an isolated GFAP × sex interaction on longitudinal change in middle temporal gyrus volume (*P*-interaction = 0.03). Post hoc analyses showed that the association of GFAP with faster declines in middle temporal gyrus volume was much stronger among men than it was among women (Supplemental Tables 4 and 5). Sex did not modify the association of Aβ_42/40_, pTau-181, and NfL with brain volume change. However, there was evidence for effect modification by sex on cross-sectional associations for each of these biomarkers (see Supplemental Table 4).

There was a significant pTau181 × *APOE*ε4 interaction on longitudinal change in parahippocampal gyrus volume (*P*-interaction = 0.02). The association of higher pTau-181 with faster parahippocampal gyrus volume loss was considerably stronger among *APOE*ε4-negative participants compared to *APOE*ε4-positive participants (Supplemental Tables 5 and 6). Modification effects of *APOE*ε4 status on brain volume change were not observed for Aβ_42/40_, GFAP, or NfL (Supplemental Table 6).

Amyloid status (defined by plasma Aβ_42/40_) modified the association between baseline pTau-181 and longitudinal brain volume changes (ventricular volume: *P*-interaction = 0.001; hippocampal volume: *P*-interaction = 0.04; total brain volume: *P*-interaction = 0.05; Supplemental Table 7). Post hoc analyses demonstrated that among those with higher Aβ burden (low Aβ_42/40_), higher baseline pTau-181 was associated with faster ventricular volume increase; however, no relationship was observed among those with lower Aβ burden (high Aβ_42/40_) (Supplemental Table 5 and 7; Fig. 2b). Surprisingly, among those with lower Aβ burden (high Aβ_42/40_), higher baseline pTau-181 was associated with accelerated declines in total brain volume and hippocampal volume, while no association was observed among those with higher Aβ burden (Supplemental Tables 5 and 7; Fig. 2b). We further show that age-related brain volume trajectories by age group vary by pTau-181 concentration and amyloid status (Supplemental Fig. 1).

### Baseline plasma biomarkers and cognitive performance

Participant characteristics are described in Table 1 (Aβ_42/40_, GFAP, and NfL: N = 674, mean age = 71.2 ± 10.1, 54.2% female, 68.6% White; pTau-181 subset: N = 602, mean age = 70.0 ± 10.6, 55.0% female, 65.6% White). Follow up time was 5.7 years (SD = 3.2) for Aβ_42/40_, GFAP, and NfL and 5.6 years (SD = 3.3) for pTau-181. Fifty-seven (8.5%) participants developed cognitive impairment during follow-up.

**Fig. 4:**
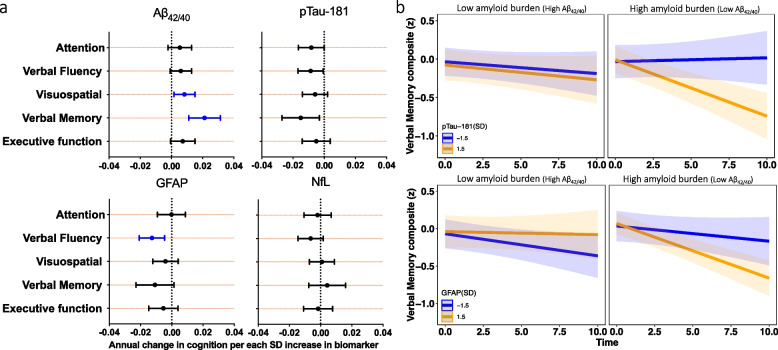
Association between baseline plasma biomarkers and longitudinal cognitive change. **a**. Results are derived from linear mixed effect models adjusted for baseline age, sex, race, education level, and estimated glomerular filtration rate (eGFR), and the interaction between covariates and time. Estimates, derived from the plasma biomarker × time interaction terms, represent the difference in annual change in cognition (β estimates) per standard deviation increase in baseline biomarker. Blue bars reflect significant associations between plasma biomarkers and cognition by domain at an FDR corrected threshold (*P* < 0.05). **b**. Results are derived from separate linear mixed effect models that examined Aβ_42/40_ as a moderator of the association between plasma biomarkers and longitudinal cognition (models were also adjusted for baseline age, sex, race, education level, eGFR, and the interaction between covariates and time). Each figure (blue lines: low biomarker [e.g., pTau-181 or GFAP] or -1.5 standard deviations below the mean; orange lines: high biomarker [e.g., pTau-181 or GFAP] or + 1.5 standard deviations above the mean) shows the marginal effects for the following interaction terms pTau-181 × time × Aβ_42/40_ status or GFAP × time × Aβ_42/40_ status on verbal memory. *Abbreviations*: Aβ: amyloid-β; GFAP: glial fibrillary acidic protein; NfL: neurofilament light chain; pTau-181: tau phosphorylated at threonine-181.

Lower Aβ_42/40_ was associated with higher verbal memory scores (*P* = 0.01), but was not related to baseline attention, executive function, verbal fluency, and visuospatial abilities. Similarly, pTau-181, GFAP, and NfL and were not associated with cognition in cross-sectional analyses (Supplemental Table 8; Supplemental Table 9 [model without eGFR as a covariate]; Supplemental Table 10 [full model]). In longitudinal analyses, lower Aβ_42/40_ was associated with faster declines in verbal memory (*P* < 0.0001) and visuospatial performance (*P* = 0.02), while higher GFAP was associated with accelerated declines in verbal fluency (*P* = 0.002; Fig. 4a) after correcting for multiple comparisons. pTau-181 and NfL levels were not associated with changes in cognition (Supplemental Table 8). The results were similar in sensitivity analyses that did not adjust for eGFR (Supplementary Table 9).

Consistent with our brain volume analyses, examination of effect modification by sex found an isolated GFAP × sex interaction on change in visuospatial skills (*P*-interaction = 0.01; Supplemental Table 11. Among men, higher baseline GFAP was associated with faster declines in visuospatial performance, but this was not the case for women (Supplemental Table 11 and 12).

*APOE*ε4 status did not modify the association between baseline plasma biomarkers and longitudinal change in cognitive performance. However, cross-sectional analyses did show evidence for *APOE*ε4 as a modifier of the cross-sectional associations of Aβ_42/40_ and NfL with measures of attention (Supplemental Table 13).

Amyloid status (defined by Aβ_42/40_) modified the associations of GFAP and pTau-181 with longitudinal change in cognitive performance (GFAP: *P*-interaction = 0.02; pTau-181: *P*-interaction = 0.03; Supplemental Tables 12 and 14). Post hoc analyses demonstrated that among participants with high Aβ burden (low Aβ_42/40_), higher baseline GFAP and pTau-181 were associated with steeper declines in verbal memory, while no associations were observed among those with a low Aβ burden (Fig. 4b). We further show that age-related verbal memory trajectories vary by pTau-181and GFAP concentration and amyloid status (Supplemental Fig. 1).

## Discussion

The present study examined whether plasma markers of AD pathology (Aβ_42/40_, pTau-181), astrogliosis (GFAP), and neuronal injury (NfL) predict longitudinal changes in brain volume and cognitive performance in a sample of cognitively unimpaired adults over a median follow-up of five to six years. We found that higher plasma pTau-181 was a robust predictor of brain volume loss, particularly in gray matter structures, while higher plasma GFAP showed a strong association with longitudinal ventricular enlargement and accelerated declines in verbal fluency. Although plasma Aβ_42/40_ and NfL were not associated with brain volumetric changes, lower plasma Aβ_42/40_ (indicative of greater Aβ burden) was associated with faster declines in verbal memory and visuospatial performance. Overall, our results suggest that higher pTau-181 and GFAP are indicative of future brain volume loss in cognitively normal older adults, while Aβ_42/40_ and GFAP levels are associated with faster subsequent cognitive declines in specific cognitive domains.

Given that declines in brain volume typically precede cognitive impairment [50], early predictors of brain atrophy may aid in identifying individuals at risk for age-related cognitive decline and cognitive impairment, including dementia. The finding that plasma pTau-181 was associated with steeper longitudinal declines in brain volume aligns with a previous study that found baseline pTau-181 to be associated with greater brain atrophy in cognitively unimpaired individuals [18]. In contrast to other studies [7, 17] which have examined both participants with and without cognitive impairment, we found that higher abundance of plasma NfL was not associated with faster subsequent declines in brain volume. One reason for this discrepancy may be disease stage. Given our focus on cognitively unimpaired older adults, participants may not be advanced enough from a neuropathological perspective to detect significant associations with a marker of neuronal injury. Previous work has demonstrated that plasma NfL does not increase in those with AD compared to healthy controls until approximately 10 years before dementia diagnosis [51]. In the present study, only 8.5% of participants went on to develop any type of dementia or mild cognitive impairment during the follow-up period. Increases in plasma NfL are thought to coincide with brain atrophy and occur after elevations in pTau-181, Aβ_42_, and GFAP [50, 52, 53]. The lack of NfL associations with brain volume change in the context of associations of pTau-181 and GFAP with brain volume change suggests that, among cognitively unimpaired older adults, NfL is comparatively less informative as a prognostic indicator of neurodegeneration.

The lack of an association between Aβ_42/40_ levels and subsequent brain changes is surprising, particularly in the context of the robust pTau-181 associations. In a recent analysis using the same (BLSA) cohort, we found that Aβ_42/40_ predicted amyloid PET status with good – but less than optimal – levels of accuracy (AUC = 0.72), with pTau-181 showing similar accuracy for prediction of amyloid status (AUC = 0.72) [12]. The divergent associations with brain volume loss despite similar predictive accuracy for cortical amyloid suggest that plasma pTau-181, in addition to acting as a measure of amyloid status [54], captures a broader range of neuropathological processes linked to neurodegeneration compared to plasma Aβ_42/40_ at this point in the disease process [18]. There are at least three additional explanations for the null association between plasma Aβ_42/40_ and brain volume. First, accuracy of the Aβ_42/40_ quantification may be limited by assay-specific factors, as the Simoa Aβ_42/40_ assay has been shown to be inferior to mass spectrometry and other immune-assays for prediction of cortical Aβ [55, 56]. Second, plasma Aβ_42/40_ has a limited dynamic range with a group level difference between healthy control and AD patients of only 10–20%, making discrimination between AD and non-AD challenging, perhaps even more so among cognitively normal individuals [50, 57]. Lastly, the accumulation of Aβ_42_ peptides, which has been proposed to be an initiating factor in AD, may be too distal in the disease processes from the neurodegenerative processes underlying brain atrophy [12].

In the current study, we found that lower plasma Aβ_42/40_ and higher GFAP were associated with faster declines in verbal memory and fluency, respectively, over approximately 6 years. The association of Aβ_42/40_ with accelerated decline in verbal memory supports the clinical utility and specificity of this biomarker in asymptomatic AD. The association of higher GFAP with faster declines in verbal fluency aligns with prior studies that suggest astrogliosis promotes synaptic dysfunction and subsequent cognitive impairment [58]. While the association of Aβ_42/40_ and GFAP with cognitive decline has been reported previously, the lack of association of NfL and pTau-181 with cognitive decline runs counter to previous findings [23]. Despite finding that higher baseline pTau-181 was associated with accelerated declines in temporal lobe brain volume, pTau-181 was not associated with cognitive decline. Given that soluble pTau-181 levels are known to rise prior to significant increases in cortical tau [52], and cortical tau is strongly associated with cognitive decline [59], associations of plasma pTau-181 with cognitive decline may be observed only at a later disease stage or with a longer follow-up period. Notably, we did see nonsignificant trends in pTau-181 associations with cognitive change, suggesting that higher levels may relate to declines in several cognitive domains.

While only 8.5% of participants went on to develop mild cognitive impairment or dementia during the follow-up period of the current study, 40% of participants were estimated to be amyloid positive at the time plasma biomarkers were measured based on plasma Aβ_42/40_ levels. We found that amyloid-positive status – an indicator of the presence of AD pathology – modified the association of plasma pTau-181 with brain volume loss such that higher pTau-181 was more strongly associated with increased ventricular volume among those with higher Aβ burden. In contrast to these results, we also found that higher pTau-181 was more strongly associated with accelerated declines in total brain and hippocampal volume among participants with a lower Aβ burden. The reason for this seemingly contradictory finding is unknown. However, these results suggest that the association of pTau-181 with total brain and hippocampal volume loss may be magnified early in the course of AD pathogenesis (ahead of any major elevations in cortical amyloid), whereas pTau-181 and GFAP are associated with faster declines in verbal memory later in the course of AD pathogenesis, in the context of high Aβ burden.

This study has several strengths, including a large sample, the availability of longitudinal outcome data from a comprehensive neuropsychological examination and repeated neuroimaging assessments on a 3T MRI, and the focus on cognitively unimpaired (asymptomatic) older adults. Despite these strengths, the current study has several limitations. First, the BLSA is a relatively healthy sample and mean level of education in the present sample was 17 years. While the sample is relatively large and diverse, it is not sufficiently powered for stratified analyses to determine whether findings are consistent across included racial and ethnic groups. Therefore, the findings derived from the present study may not be generalizable to the broader population. Second, only a small percentage of participants progressed to cognitive impairment, which may have limited our ability to detect significant associations among some plasma biomarkers (e.g., NfL) and brain volume. Finally, the present study did not include measurement of the pTau-217 isoform, which compared to pTau-181, has demonstrated greater accuracy for prediction of cortical AD pathology [60, 61]. Further, the addition of pTau-217 may have enabled us to detect differences in brain volume and cognition as a function of sex, as prior work has demonstrated that higher pTau-217 is related to declines in verbal memory and brain atrophy for cognitively unimpaired women but not men [62].

## Conclusions

The current study demonstrates that plasma biomarkers of AD pathology (pTau-181) and astrogliosis (GFAP), but not neuronal injury (NfL), may serve as markers of future brain atrophy and cognitive decline among cognitively unimpaired older adults in the community. In addition to demonstrating that these plasma biomarkers are associated with future neurodegenerative and cognitive trajectories, we show that the extent to which GFAP and pTau-181 predict brain atrophy and cognitive decline depends on the burden of brain amyloid.

### Supplementary Information

**Supplementary Material 1.**

**Supplementary Material 2.**

**Supplementary Material 3.**

## Data Availability

No datasets were generated or analysed during the current study.

## Abbreviations

AD: Alzheimer’s disease
Aβ: Amyloid-β
BLSA: Baltimore Longitudinal Study of Aging
CVs: Coefficients of variation
eGFR: Estimated glomerular filtration rate
FDR: False discovery rate
GFAP: Glial fibrillary acidic protein
ICV: Total intracranial volume
LME: Linear mixed effects
MPRAGE: Magnetization-prepared rapid gradient echo
MUSE: Multi-atlas Region Segmentation Utilizing Ensembles
N4PE: Neurology 4-Plex E
NfL: Neurofilament light chain
NFT: Neurofibrillary tangles
PCR: Polymerase chain reaction
PET: Positron emission tomography
PiB: ^11^C-Pittsburgh compound-B
pTau-181: Tau phosphorylated at threonine-181
ROIs: Regions of interest
VBM: Voxel-based morphometry
