# Supplementary material for: Alzheimer’s and neurodegenerative disease biomarkers in blood predict brain atrophy and cognitive decline
Source: Alzheimers Res Ther. 2024 Apr 30;16:94. doi: 10.1186/s13195-024-01459-y (PMC11059745; doi:10.1186/s13195-024-01459-y)
Supplement: Supplementary file 1 — Supplementary Material 1. [file 13195_2024_1459_MOESM1_ESM.docx]

**Alzheimer’s and neurodegenerative disease biomarkers in blood predict brain atrophy and cognitive decline**

Table of Contents

[Supplemental Table 1. Associations between baseline plasma biomarkers and brain volume 2](#_Toc163814590)

[Supplemental Table 2. Associations between baseline plasma biomarkers and brain volume (without eGFR as a covariate) 4](#_Toc163814591)

[Supplemental Table 3. Associations between baseline plasma biomarkers and brain volume (full model results) 6](#_Toc163814592)

[Supplemental Table 4. The effects of sex on the relationship between baseline plasma biomarkers and brain volume 7](#_Toc163814593)

[Supplemental Table 5. Modifier-specific (sex, *APOE*ε4 status, and Aβ_42/40_ status) estimates of the relationship between baseline plasma biomarkers and brain volume change 9](#_Toc163814594)

[Supplemental Table 6. The effects of *APOE*ε4 status on the relationship between baseline plasma biomarkers and brain volume 10](#_Toc163814595)

[Supplemental Table 7. The effects of Aβ_42/40_ status on the relationship between baseline plasma biomarkers and brain volume 12](#_Toc163814596)

[Supplemental Table 8. Associations between baseline plasma biomarkers and cognitive performance 13](#_Toc163814597)

[Supplemental Table 9. Associations between baseline plasma biomarkers and cognitive performance (without eGFR as a covariate) 14](#_Toc163814598)

[Supplemental Table 10. Associations between baseline plasma biomarkers and cognitive performance (full model results) 15](#_Toc163814599)

[Supplemental Table 11. The effects of sex on the relationship between baseline plasma biomarkers and cognitive performance 16](#_Toc163814600)

[Supplemental Table 12. Modifier-specific (sex and Aβ_42/40_ status) estimates of the relationship between baseline plasma biomarkers and change in cognitive performance 17](#_Toc163814601)

[Supplemental Table 13. The effects of *APOE*ε4 status on the relationship between baseline plasma biomarkers and cognitive performance 18](#_Toc163814602)

[Supplemental Table 14. The effects of Aβ_42/40_ status on the relationship between baseline plasma biomarkers and cognitive performance 19](#_Toc163814603)

[Supplemental Figure 1. Effect of baseline plasma biomarkers on age-related trajectories of brain volume and verbal memory, stratified by Aβ_42/40_ status 20](#_Toc163814604)

**Supplemental Tables**

# Supplemental Table 1. Associations between baseline plasma biomarkers and brain volume

|  |  | Cross-sectional effects | | | Longitudinal effects | | |
| --- | --- | --- | --- | --- | --- | --- | --- |
| Biomarker | ROI volumes | β | SE | *P* | β | SE | *P* |
| Aβ_42/40_ | Total brain | 0.0002 | 0.01 | 0.99 | 0.002 | 0.001 | 0.19 |
|  | White matter | -0.001 | 0.02 | 0.95 | 0.001 | 0.001 | 0.23 |
|  | Gray matter | 0.003 | 0.02 | 0.86 | 0.002 | 0.002 | 0.35 |
|  | Ventricular CSF | -0.003 | 0.03 | 0.93 | -0.002 | 0.002 | 0.37 |
|  | Superior temporal gyrus | 0.03 | 0.03 | 0.37 | 0.002 | 0.002 | 0.30 |
|  | Middle temporal gyrus | -0.02 | 0.03 | 0.48 | 0.003 | 0.002 | 0.16 |
|  | Inferior temporal gyrus | -0.01 | 0.03 | 0.70 | 0.002 | 0.002 | 0.25 |
|  | Hippocampus | -0.01 | 0.03 | 0.87 | 0.003 | 0.002 | 0.18 |
|  | Parahippocampal gyrus | -0.002 | 0.03 | 0.95 | 0.001 | 0.002 | 0.55 |
|  | Entorhinal cortex | 0.02 | 0.03 | 0.51 | 0.002 | 0.003 | 0.50 |
|  | Amygdala | 0.01 | 0.03 | 0.71 | 0.002 | 0.003 | 0.42 |
|  |  | β | SE | *P* | β | SE | *P* |
| Ptau-181 | Total brain | 0.01 | 0.01 | 0.37 | -0.003 | 0.002 | 0.05 |
|  | White matter | 0.04 | 0.02 | 0.09 | -0.001 | 0.001 | 0.63 |
|  | Gray matter | -0.01 | 0.02 | 0.47 | -0.01 | 0.002 | 0.005* |
|  | Ventricular CSF | 0.01 | 0.04 | 0.77 | 0.01 | 0.002 | 0.007* |
|  | Superior temporal gyrus | 0.02 | 0.03 | 0.57 | -0.01 | 0.002 | 0.001* |
|  | Middle temporal gyrus | -0.03 | 0.03 | 0.32 | -0.01 | 0.002 | 0.001* |
|  | Inferior temporal gyrus | -0.12 | 0.03 | <0.0001* | -0.01 | 0.003 | 0.005* |
|  | Hippocampus | -0.06 | 0.03 | 0.09 | -0.01 | 0.003 | 0.049 |
|  | Parahippocampal gyrus | 0.03 | 0.03 | 0.39 | -0.01 | 0.003 | 0.002* |
|  | Entorhinal cortex | -0.01 | 0.03 | 0.78 | 0.001 | 0.004 | 0.83 |
|  | Amygdala | -0.003 | 0.03 | 0.93 | -0.01 | 0.003 | 0.009* |
|  |  | β | SE | *P* | β | SE | *P* |
| GFAP | Total brain | -0.01 | 0.01 | 0.54 | 0.001 | 0.001 | 0.69 |
|  | White matter | -0.02 | 0.02 | 0.34 | 0.001 | 0.001 | 0.59 |
|  | Gray matter | -0.03 | 0.02 | 0.11 | -0.002 | 0.002 | 0.30 |
|  | Ventricular CSF | 0.10 | 0.04 | 0.01 | 0.01 | 0.002 | <0.0001* |
|  | Superior temporal gyrus | -0.01 | 0.03 | 0.87 | -0.002 | 0.002 | 0.24 |
|  | Middle temporal gyrus | -0.02 | 0.03 | 0.54 | -0.003 | 0.002 | 0.16 |
|  | Inferior temporal gyrus | 0.001 | 0.03 | 0.97 | -0.003 | 0.003 | 0.26 |
|  | Hippocampus | 0.02 | 0.03 | 0.62 | -0.01 | 0.003 | 0.06 |
|  | Parahippocampal gyrus | 0.02 | 0.03 | 0.54 | -0.003 | 0.003 | 0.27 |
|  | Entorhinal cortex | 0.04 | 0.03 | 0.20 | -0.005 | 0.003 | 0.16 |
|  | Amygdala | 0.05 | 0.03 | 0.13 | -0.01 | 0.003 | 0.03 |
|  |  | β | SE | *P* | β | SE | *P* |
| NfL | Total brain | -0.01 | 0.01 | 0.40 | 0.004 | 0.001 | 0.01 |
|  | White matter | 0.02 | 0.02 | 0.35 | 0.003 | 0.001 | 0.02 |
|  | Gray matter | -0.04 | 0.02 | 0.05 | 0.004 | 0.002 | 0.04 |
|  | Ventricular CSF | -0.01 | 0.04 | 0.81 | -0.0002 | 0.002 | 0.95 |
|  | Superior temporal gyrus | 0.0001 | 0.04 | 1.00 | 0.003 | 0.002 | 0.18 |
|  | Middle temporal gyrus | -0.03 | 0.03 | 0.36 | 0.003 | 0.002 | 0.16 |
|  | Inferior temporal gyrus | -0.01 | 0.03 | 0.63 | 0.005 | 0.003 | 0.08 |
|  | Hippocampus | 0.01 | 0.04 | 0.76 | -0.0002 | 0.003 | 0.94 |
|  | Parahippocampal gyrus | -0.003 | 0.03 | 0.92 | 0.004 | 0.003 | 0.15 |
|  | Entorhinal cortex | 0.04 | 0.03 | 0.26 | -0.002 | 0.003 | 0.55 |
|  | Amygdala | -0.01 | 0.04 | 0.82 | 0.001 | 0.003 | 0.73 |

Note: Results are derived from linear mixed effect models adjusted for baseline age, sex, race, education level, estimated glomerular filtration rate (eGFR), total intracranial volume at age 70, and time-covariate interactions. Cross-sectional β values reflect the difference in brain volume per standard deviation (SD) increase in biomarker. Longitudinal β values reflect the difference in annual change in brain volume per SD increase in biomarker. Cross-sectional effects reflect the main effect of each plasma biomarker, whereas longitudinal effects reflect plasma biomarker × time interactions derived from the same model. *Remains significant after 0.05 FDR correction. *Abbreviations*: Aβ: amyloid-β; CSF: cerebral spinal fluid; GFAP: glial fibrillary acidic protein; NfL: neurofilament light chain; ROI: region of interest; pTau-181: tau phosphorylated at threonine-181; SE: standard error.

# Supplemental Table 2. Associations between baseline plasma biomarkers and brain volume (without eGFR as a covariate)

|  |  | Cross-sectional effects | | | Longitudinal effects | | |
| --- | --- | --- | --- | --- | --- | --- | --- |
| Biomarker | ROI volumes | β | SE | *P* | β | SE | *P* |
| Aβ_42/40_ | Total brain | 0.0005 | 0.01 | 0.97 | 0.002 | 0.001 | 0.18 |
|  | White matter | -0.001 | 0.02 | 0.94 | 0.001 | 0.001 | 0.22 |
|  | Gray matter | 0.004 | 0.02 | 0.82 | 0.002 | 0.002 | 0.36 |
|  | Ventricular CSF | -0.003 | 0.03 | 0.92 | -0.002 | 0.002 | 0.39 |
|  | Superior temporal gyrus | 0.03 | 0.03 | 0.34 | 0.002 | 0.002 | 0.35 |
|  | Middle temporal gyrus | -0.02 | 0.03 | 0.50 | 0.003 | 0.002 | 0.18 |
|  | Inferior temporal gyrus | -0.01 | 0.03 | 0.70 | 0.002 | 0.002 | 0.27 |
|  | Hippocampus | -0.01 | 0.03 | 0.86 | 0.003 | 0.002 | 0.17 |
|  | Parahippocampal gyrus | -0.001 | 0.03 | 0.97 | 0.001 | 0.002 | 0.58 |
|  | Entorhinal cortex | 0.02 | 0.03 | 0.49 | 0.002 | 0.003 | 0.56 |
|  | Amygdala | 0.01 | 0.03 | 0.70 | 0.002 | 0.003 | 0.44 |
|  |  | β | SE | *P* | β | SE | *P* |
| Ptau-181 | Total brain | 0.01 | 0.01 | 0.42 | -0.003 | 0.001 | 0.06 |
|  | White matter | 0.04 | 0.02 | 0.06 | -0.001 | 0.001 | 0.51 |
|  | Gray matter | -0.02 | 0.02 | 0.34 | -0.01 | 0.002 | 0.01* |
|  | Ventricular CSF | 0.01 | 0.04 | 0.88 | 0.01 | 0.002 | 0.02* |
|  | Superior temporal gyrus | 0.001 | 0.03 | 0.98 | -0.01 | 0.002 | 0.01* |
|  | Middle temporal gyrus | -0.04 | 0.03 | 0.19 | -0.01 | 0.002 | 0.003* |
|  | Inferior temporal gyrus | -0.12 | 0.03 | <0.0001* | -0.01 | 0.003 | 0.02* |
|  | Hippocampus | -0.05 | 0.03 | 0.13 | -0.01 | 0.003 | 0.05 |
|  | Parahippocampal gyrus | 0.02 | 0.03 | 0.51 | -0.01 | 0.003 | 0.01* |
|  | Entorhinal cortex | -0.01 | 0.03 | 0.67 | 0.002 | 0.003 | 0.49 |
|  | Amygdala | -0.01 | 0.03 | 0.80 | -0.01 | 0.003 | 0.04 |
|  |  | β | SE | *P* | β | SE | *P* |
| GFAP | Total brain | -0.01 | 0.01 | 0.51 | 0.001 | 0.001 | 0.70 |
|  | White matter | -0.02 | 0.02 | 0.35 | 0.001 | 0.001 | 0.62 |
|  | Gray matter | -0.03 | 0.02 | 0.09 | -0.002 | 0.002 | 0.33 |
|  | Ventricular CSF | 0.10 | 0.04 | 0.01 | 0.01 | 0.002 | <0.0001* |
|  | Superior temporal gyrus | -0.01 | 0.03 | 0.70 | -0.002 | 0.002 | 0.33 |
|  | Middle temporal gyrus | -0.02 | 0.03 | 0.48 | -0.003 | 0.002 | 0.19 |
|  | Inferior temporal gyrus | 0.002 | 0.03 | 0.95 | -0.003 | 0.003 | 0.28 |
|  | Hippocampus | 0.02 | 0.03 | 0.58 | -0.01 | 0.003 | 0.05 |
|  | Parahippocampal gyrus | 0.02 | 0.03 | 0.58 | -0.003 | 0.003 | 0.31 |
|  | Entorhinal cortex | 0.04 | 0.03 | 0.22 | -0.004 | 0.003 | 0.21 |
|  | Amygdala | 0.05 | 0.03 | 0.15 | -0.01 | 0.003 | 0.03 |
|  |  | β | SE | *P* | β | SE | *P* |
| NfL | Total brain | -0.01 | 0.01 | 0.33 | 0.003 | 0.001 | 0.02 |
|  | White matter | 0.02 | 0.02 | 0.33 | 0.003 | 0.001 | 0.02 |
|  | Gray matter | -0.04 | 0.02 | 0.02 | 0.004 | 0.002 | 0.04 |
|  | Ventricular CSF | -0.01 | 0.04 | 0.89 | -0.0004 | 0.002 | 0.86 |
|  | Superior temporal gyrus | -0.02 | 0.03 | 0.61 | 0.003 | 0.002 | 0.10 |
|  | Middle temporal gyrus | -0.04 | 0.03 | 0.25 | 0.004 | 0.002 | 0.12 |
|  | Inferior temporal gyrus | -0.01 | 0.03 | 0.69 | 0.004 | 0.003 | 0.08 |
|  | Hippocampus | 0.02 | 0.03 | 0.66 | -0.001 | 0.003 | 0.82 |
|  | Parahippocampal gyrus | -0.01 | 0.03 | 0.81 | 0.004 | 0.003 | 0.12 |
|  | Entorhinal cortex | 0.03 | 0.03 | 0.33 | -0.001 | 0.003 | 0.76 |
|  | Amygdala | -0.01 | 0.03 | 0.72 | 0.002 | 0.003 | 0.64 |

Note: Results are derived from linear mixed effect models adjusted for baseline age, sex, race, education level, total intracranial volume at age 70, and time-covariate interactions. Cross-sectional β values reflect the difference in brain volume per standard deviation (SD) increase in biomarker. Longitudinal β values reflect the difference in annual change in brain volume per SD increase in biomarker. Cross-sectional effects reflect the main effect of each plasma biomarker, whereas longitudinal effects reflect plasma biomarker × time interactions derived from the same model. *Remains significant after 0.05 FDR correction. *Abbreviations*: Aβ: amyloid-β; CSF: cerebral spinal fluid; eGFR: estimated glomerular filtration rate; GFAP: glial fibrillary acidic protein; NfL: neurofilament light chain; ROI: region of interest; pTau-181: tau phosphorylated at threonine-181; SE: standard error.

# Supplemental Table 3. Associations between baseline plasma biomarkers and brain volume (full model results)

*(See excel spreadsheet)*

# Supplemental Table 4. The effects of sex on the relationship between baseline plasma biomarkers and brain volume

|  |  | Cross-sectional effects | | | Longitudinal effects | | | | |
| --- | --- | --- | --- | --- | --- | --- | --- | --- | --- |
|  | ROI | β | SE | *P* | β | SE | | | *P* |
| Aβ_42/40_ × sex | Total brain | -0.004 | 0.03 | 0.88 | 0.001 | 0.002 | | | 0.61 |
|  | White matter | -0.02 | 0.04 | 0.60 | 0.001 | 0.002 | | | 0.55 |
|  | Gray matter | 0.02 | 0.03 | 0.63 | 0.0003 | 0.003 | | | 0.94 |
|  | Ventricular CSF | -0.01 | 0.07 | 0.84 | -0.002 | 0.004 | | | 0.66 |
|  | Superior temporal gyrus | 0.14 | 0.06 | 0.02 | 0.004 | 0.003 | | | 0.28 |
|  | Middle temporal gyrus | 0.02 | 0.05 | 0.77 | 0.003 | 0.004 | | | 0.38 |
|  | Inferior temporal gyrus | 0.07 | 0.05 | 0.16 | 0.002 | 0.004 | | | 0.69 |
|  | Hippocampus | -0.03 | 0.06 | 0.65 | -0.002 | 0.005 | | | 0.70 |
|  | Parahippocampal gyrus | 0.05 | 0.06 | 0.35 | -0.005 | 0.005 | | | 0.31 |
|  | Entorhinal cortex | -0.02 | 0.06 | 0.69 | -0.001 | 0.01 | | | 0.91 |
|  | Amygdala | -0.01 | 0.06 | 0.84 | 0.0004 | 0.01 | | | 0.95 |
|  |  | β | SE | *P* | β | SE | | | *P* |
| pTau-181 × sex | Total brain | -0.02 | 0.03 | 0.54 | -0.004 | 0.003 | | | 0.12 |
|  | White matter | -0.002 | 0.04 | 0.96 | -0.003 | 0.003 | | | 0.23 |
|  | Gray matter | -0.005 | 0.03 | 0.89 | -0.01 | 0.004 | | | 0.09 |
|  | Ventricular CSF | -0.04 | 0.07 | 0.57 | 0.0003 | 0.004 | | | 0.95 |
|  | Superior temporal gyrus | -0.03 | 0.06 | 0.65 | -0.01 | 0.004 | | | 0.08 |
|  | Middle temporal gyrus | -0.02 | 0.06 | 0.68 | -0.01 | 0.004 | | | 0.15 |
|  | Inferior temporal gyrus | -0.11 | 0.05 | 0.04 | -0.01 | 0.005 | | | 0.12 |
|  | Hippocampus | -0.04 | 0.06 | 0.48 | -0.01 | 0.005 | | | 0.09 |
|  | Parahippocampal gyrus | -0.06 | 0.06 | 0.30 | -0.004 | 0.005 | | | 0.40 |
|  | Entorhinal cortex | 0.03 | 0.06 | 0.59 | -0.01 | 0.01 | | | 0.35 |
|  | Amygdala | -0.03 | 0.06 | 0.59 | -0.001 | 0.01 | | | 0.86 |
|  |  | β | SE | *P* | β | | SE | *P* | |
| GFAP × sex | Total brain | -0.03 | 0.02 | 0.17 | -0.001 | | 0.003 | 0.68 | |
|  | White matter | -0.06 | 0.04 | 0.11 | -0.001 | | 0.002 | 0.83 | |
|  | Gray matter | -0.02 | 0.03 | 0.51 | -0.004 | | 0.004 | 0.30 | |
|  | Ventricular CSF | 0.05 | 0.06 | 0.41 | 0.001 | | 0.004 | 0.88 | |
|  | Superior temporal gyrus | -0.06 | 0.06 | 0.29 | -0.002 | | 0.004 | 0.60 | |
|  | Middle temporal gyrus | -0.01 | 0.05 | 0.85 | -0.01 | | 0.004 | 0.03 | |
|  | Inferior temporal gyrus | -0.02 | 0.05 | 0.73 | -0.01 | | 0.004 | 0.07 | |
|  | Hippocampus | -0.03 | 0.06 | 0.56 | -0.004 | | 0.005 | 0.38 | |
|  | Parahippocampal gyrus | -0.02 | 0.05 | 0.72 | -0.0005 | | 0.005 | 0.92 | |
|  | Entorhinal cortex | 0.04 | 0.06 | 0.49 | -0.01 | | 0.01 | 0.09 | |
|  | Amygdala | -0.01 | 0.06 | 0.82 | -0.004 | | 0.01 | 0.48 | |
|  |  | β | SE | *P* | β | SE | | | *P* |
| NfL × sex | Total brain | -0.04 | 0.02 | 0.12 | -0.002 | 0.002 | | | 0.51 |
|  | White matter | -0.06 | 0.04 | 0.08 | 0.001 | 0.002 | | | 0.73 |
|  | Gray matter | -0.04 | 0.03 | 0.26 | -0.003 | 0.004 | | | 0.48 |
|  | Ventricular CSF | 0.07 | 0.06 | 0.24 | -0.004 | 0.004 | | | 0.28 |
|  | Superior temporal gyrus | -0.14 | 0.06 | 0.02 | -0.001 | 0.004 | | | 0.74 |
|  | Middle temporal gyrus | -0.04 | 0.05 | 0.47 | -0.01 | 0.004 | | | 0.09 |
|  | Inferior temporal gyrus | -0.11 | 0.05 | 0.02 | -0.01 | 0.004 | | | 0.15 |
|  | Hippocampus | -0.01 | 0.06 | 0.84 | -0.001 | 0.005 | | | 0.76 |
|  | Parahippocampal gyrus | -0.02 | 0.05 | 0.71 | -0.001 | 0.005 | | | 0.80 |
|  | Entorhinal cortex | 0.01 | 0.06 | 0.88 | -0.001 | 0.01 | | | 0.89 |
|  | Amygdala | -0.07 | 0.06 | 0.23 | 0.003 | 0.01 | | | 0.60 |

Note. Results are derived from linear mixed effect models that examined interactions between plasma biomarkers and sex on brain volume (adjusted for baseline age, sex, race, education level, estimated glomerular filtration rate (eGFR), total intracranial volume at age 70, and time-covariate interactions).

Cross-sectional effects reflect whether the association between each plasma biomarker and brain volume varies by sex. Longitudinal effects reflect whether the association between each plasma biomarker and changes in brain volume vary by sex. Cross-sectional effects reflect plasma biomarker × sex interactions, whereas longitudinal effects reflect plasma biomarker × sex × time interactions from the same model. No associations survived FDR correction. *Abbreviations*: Aβ: amyloid-β; CSF: cerebral spinal fluid; GFAP: glial fibrillary acidic protein; NfL: neurofilament light chain; ROI: region of interest; pTau-181: tau phosphorylated at threonine-181; SE: standard error.

# Supplemental Table 5. Modifier-specific (sex, *APOE*ε4 status, and Aβ_42/40_ status) estimates of the relationship between baseline plasma biomarkers and brain volume change

| Significant 3-way interactions | | Modifier-specific estimates | | | | | |
| --- | --- | --- | --- | --- | --- | --- | --- |
|  | | Male | | | Female | | |
|  |  | β | SE | *P* | β | SE | *P* |
| GFAP × sex × time | Middle temporal gyrus | -0.01 | 0.003 | 0.01 | -0.00002 | 0.003 | 1.00 |
|  |  | *APOE*ε4-negative | | | *APOE*ε4-positive | | |
|  |  | β | SE | *P* | β | SE | *P* |
| pTau-181 × *APOE*ε4 status × time | Parahippocampal gyrus | -0.01 | 0.003 | 0.0001 | 0.001 | 0.01 | 0.90 |
|  |  | Low Aβ_42/40_ | | | High Aβ_42/40_ | | |
|  |  | β | SE | *P* | β | SE | *P* |
| pTau-181 × Aβ_42/40_ status × time | Total Brain | -0.0003 | 0.002 | 0.87 | -0.01 | 0.002 | 0.01 |
| pTau-181 × Aβ_42/40_ status × time | Ventricular CSF | 0.01 | 0.003 | <0.0001 | -0.001 | 0.003 | 0.74 |
| pTau-181 × Aβ_42/40_ status × time | Hippocampus | -0.001 | 0.004 | 0.86 | -0.01 | 0.004 | 0.004 |

Note. β values reflect modifier-specific estimates that are derived from the same linear mixed effect models that examined interactions between plasma biomarkers and sex, *APOE*ε4 status, and Aβ_42/40_ status on brain volume (adjusted for baseline age, sex, race, education level, estimated glomerular filtration rate (eGFR), total intracranial volume at age 70, and time-covariate interactions). Only longitudinal effects are presented in the table. Longitudinal effects reflect whether the association between each plasma biomarker and changes in brain volume vary by sex, *APOE*ε4 status, or Aβ_42/40_ status. Only results from significant three-way interactions are presented. Multiple comparison correction was not completed for these analyses.

# Supplemental Table 6. The effects of *APOE*ε4 status on the relationship between baseline plasma biomarkers and brain volume

|  |  | Cross-sectional effects | | | Longitudinal effects | | | | |
| --- | --- | --- | --- | --- | --- | --- | --- | --- | --- |
|  | ROI volumes | β | SE | *P* | β | | SE | | *P* |
| Aβ_42/40_ × *APOE*ε4 | Total brain | -0.03 | 0.03 | 0.31 | -0.004 | | 0.003 | | 0.19 |
|  | White matter | -0.03 | 0.04 | 0.48 | -0.001 | | 0.003 | | 0.83 |
|  | Gray matter | -0.01 | 0.04 | 0.79 | -0.005 | | 0.004 | | 0.25 |
|  | Ventricular CSF | -0.07 | 0.08 | 0.35 | -0.005 | | 0.005 | | 0.30 |
|  | Superior temporal gyrus | -0.004 | 0.07 | 0.95 | -0.002 | | 0.004 | | 0.71 |
|  | Middle temporal gyrus | 0.01 | 0.06 | 0.87 | 0.000001 | | 0.005 | | 1.00 |
|  | Inferior temporal gyrus | 0.004 | 0.06 | 0.95 | -0.004 | | 0.01 | | 0.48 |
|  | Hippocampus | 0.04 | 0.07 | 0.55 | 0.005 | | 0.01 | | 0.40 |
|  | Parahippocampal gyrus | -0.04 | 0.07 | 0.57 | -0.001 | | 0.01 | | 0.83 |
|  | Entorhinal cortex | -0.05 | 0.07 | 0.44 | -0.01 | | 0.01 | | 0.11 |
|  | Amygdala | -0.01 | 0.07 | 0.88 | 0.004 | | 0.01 | | 0.59 |
|  |  | β | SE | *P* | β | | SE | | *P* |
| pTau-181 × *APOE*ε4 | Total brain | -0.03 | 0.03 | 0.31 | 0.01 | | 0.003 | | 0.05 |
|  | White matter | -0.02 | 0.04 | 0.57 | 0.003 | | 0.003 | | 0.30 |
|  | Gray matter | -0.05 | 0.04 | 0.21 | 0.01 | | 0.004 | | 0.07 |
|  | Ventricular CSF | 0.03 | 0.08 | 0.69 | 0.00 | | 0.005 | | 0.94 |
|  | Superior temporal gyrus | -0.08 | 0.07 | 0.29 | 0.01 | | 0.004 | | 0.13 |
|  | Middle temporal gyrus | -0.05 | 0.06 | 0.43 | 0.01 | | 0.005 | | 0.26 |
|  | Inferior temporal gyrus | -0.002 | 0.06 | 0.97 | 0.01 | | 0.01 | | 0.11 |
|  | Hippocampus | -0.01 | 0.07 | 0.94 | 0.01 | | 0.01 | | 0.15 |
|  | Parahippocampal gyrus | -0.03 | 0.07 | 0.68 | 0.01 | | 0.01 | | 0.02 |
|  | Entorhinal cortex | -0.07 | 0.07 | 0.32 | 0.003 | | 0.01 | | 0.64 |
|  | Amygdala | -0.14 | 0.07 | 0.05 | 0.002 | | 0.01 | | 0.75 |
|  |  | β | SE | *P* | β | SE | | *P* | |
| GFAP × *APOE*ε4 | Total brain | -0.03 | 0.03 | 0.34 | 0.0002 | 0.003 | | 0.95 | |
|  | White matter | -0.04 | 0.04 | 0.33 | -0.001 | 0.003 | | 0.69 | |
|  | Gray matter | -0.01 | 0.04 | 0.88 | 0.003 | 0.004 | | 0.47 | |
|  | Ventricular CSF | -0.04 | 0.07 | 0.60 | -0.002 | 0.005 | | 0.73 | |
|  | Superior temporal gyrus | -0.08 | 0.07 | 0.25 | 0.005 | 0.004 | | 0.20 | |
|  | Middle temporal gyrus | -0.02 | 0.06 | 0.72 | 0.002 | 0.004 | | 0.61 | |
|  | Inferior temporal gyrus | 0.02 | 0.06 | 0.78 | 0.01 | 0.005 | | 0.25 | |
|  | Hippocampus | 0.05 | 0.07 | 0.44 | -0.01 | 0.01 | | 0.31 | |
|  | Parahippocampal gyrus | 0.06 | 0.06 | 0.32 | 0.004 | 0.01 | | 0.48 | |
|  | Entorhinal cortex | 0.03 | 0.07 | 0.68 | 0.003 | 0.01 | | 0.61 | |
|  | Amygdala | -0.05 | 0.07 | 0.51 | 0.002 | 0.01 | | 0.80 | |
|  |  | β | SE | *P* | β | | SE | | *P* |
| NfL × *APOE*ε4 | Total brain | 0.01 | 0.03 | 0.79 | -0.003 | | 0.003 | | 0.36 |
|  | White matter | 0.04 | 0.05 | 0.34 | -0.002 | | 0.003 | | 0.54 |
|  | Gray matter | -0.02 | 0.04 | 0.64 | -0.002 | | 0.004 | | 0.62 |
|  | Ventricular CSF | -0.02 | 0.08 | 0.77 | 0.0002 | | 0.005 | | 0.97 |
|  | Superior temporal gyrus | -0.08 | 0.08 | 0.31 | 0.005 | | 0.004 | | 0.23 |
|  | Middle temporal gyrus | -0.07 | 0.07 | 0.32 | -0.00003 | | 0.005 | | 1.00 |
|  | Inferior temporal gyrus | 0.03 | 0.07 | 0.68 | 0.002 | | 0.01 | | 0.64 |
|  | Hippocampus | 0.07 | 0.08 | 0.34 | -0.002 | | 0.01 | | 0.70 |
|  | Parahippocampal gyrus | 0.12 | 0.07 | 0.10 | -0.003 | | 0.01 | | 0.59 |
|  | Entorhinal cortex | -0.03 | 0.07 | 0.69 | -0.004 | | 0.01 | | 0.55 |
|  | Amygdala | -0.07 | 0.08 | 0.39 | -0.003 | | 0.01 | | 0.63 |

Note. Results are derived from linear mixed effect models that examined interactions between plasma biomarkers and *APOE*ε4 status on brain volume (adjusted for baseline age, sex, race, education level, estimated glomerular filtration rate (eGFR), total intracranial volume at age 70, and time-covariate interactions).

Cross-sectional effects reflect whether the association between each plasma biomarker and brain volume varies by *APOE*ε4 status. Longitudinal effects reflect whether the association between each plasma biomarker and change in brain volume varies by *APOE*ε4 status. Cross-sectional effects reflect plasma biomarker × *APOE*ε4 status interactions, whereas longitudinal effects reflect plasma biomarker × *APOE*ε4 status × time interactions. No associations survived FDR correction. *Abbreviations*: Aβ: amyloid-β; CSF: cerebral spinal fluid; GFAP: glial fibrillary acidic protein; NfL: neurofilament light chain; ROI: region of interest; pTau-181: tau phosphorylated at threonine-181; SE: standard error.

# Supplemental Table 7. The effects of Aβ_42/40_ status on the relationship between baseline plasma biomarkers and brain volume

|  | |  | Cross-sectional effects | | | Longitudinal effects | | |
| --- | --- | --- | --- | --- | --- | --- | --- | --- |
|  | ROI | | β | SE | *P* | β | SE | *P* |
| pTau-181 × Aβ_42/40_ status | | Total brain | 0.002 | 0.03 | 0.95 | 0.01 | 0.003 | 0.05 |
|  | | White matter | -0.05 | 0.04 | 0.21 | 0.003 | 0.003 | 0.20 |
|  | | Gray matter | -0.01 | 0.04 | 0.81 | 0.003 | 0.004 | 0.52 |
|  | | Ventricular CSF | 0.18 | 0.07 | 0.01 | 0.01 | 0.004 | 0.001* |
|  | | Superior temporal gyrus | 0.05 | 0.07 | 0.44 | 0.001 | 0.004 | 0.87 |
|  | | Middle temporal gyrus | -0.04 | 0.06 | 0.52 | -0.002 | 0.004 | 0.68 |
|  | | Inferior temporal gyrus | -0.05 | 0.06 | 0.38 | -0.002 | 0.005 | 0.75 |
|  | | Hippocampus | -0.001 | 0.07 | 0.99 | 0.01 | 0.005 | 0.04 |
|  | | Parahippocampal gyrus | -0.04 | 0.06 | 0.53 | 0.01 | 0.01 | 0.27 |
|  | | Entorhinal cortex | -0.05 | 0.07 | 0.49 | 0.002 | 0.01 | 0.73 |
|  | | Amygdala | -0.09 | 0.07 | 0.19 | 0.004 | 0.01 | 0.50 |
|  | | | β | SE | *P* | β | SE | *P* |
| GFAP × Aβ_42/40_ status | | Total brain | 0.005 | 0.03 | 0.86 | -0.001 | 0.003 | 0.63 |
|  | | White matter | -0.03 | 0.04 | 0.51 | 0.002 | 0.002 | 0.51 |
|  | | Gray matter | 0.02 | 0.03 | 0.62 | -0.003 | 0.004 | 0.47 |
|  | | Ventricular CSF | 0.04 | 0.07 | 0.58 | 0.001 | 0.004 | 0.73 |
|  | | Superior temporal gyrus | 0.07 | 0.06 | 0.29 | -0.002 | 0.004 | 0.53 |
|  | | Middle temporal gyrus | -0.003 | 0.06 | 0.96 | -0.005 | 0.004 | 0.24 |
|  | | Inferior temporal gyrus | 0.02 | 0.05 | 0.73 | -0.005 | 0.005 | 0.30 |
|  | | Hippocampus | 0.05 | 0.06 | 0.39 | 0.002 | 0.005 | 0.74 |
|  | | Parahippocampal gyrus | 0.04 | 0.06 | 0.47 | -0.002 | 0.005 | 0.63 |
|  | | Entorhinal cortex | 0.03 | 0.06 | 0.69 | -0.004 | 0.01 | 0.56 |
|  | | Amygdala | 0.01 | 0.06 | 0.90 | -0.005 | 0.01 | 0.43 |
|  | | | β | SE | *P* | β | SE | *P* |
| NfL × Aβ_42/40_ status | | Total brain | -0.01 | 0.02 | 0.66 | -0.003 | 0.002 | 0.23 |
|  | | White matter | -0.04 | 0.04 | 0.25 | -0.001 | 0.002 | 0.82 |
|  | | Gray matter | -0.01 | 0.03 | 0.65 | -0.002 | 0.004 | 0.56 |
|  | | Ventricular CSF | 0.08 | 0.06 | 0.22 | -0.002 | 0.004 | 0.61 |
|  | | Superior temporal gyrus | 0.02 | 0.06 | 0.79 | -0.004 | 0.004 | 0.28 |
|  | | Middle temporal gyrus | -0.02 | 0.05 | 0.78 | -0.003 | 0.004 | 0.41 |
|  | | Inferior temporal gyrus | 0.0004 | 0.05 | 0.99 | -0.003 | 0.004 | 0.47 |
|  | | Hippocampus | -0.01 | 0.06 | 0.93 | 0.004 | 0.005 | 0.44 |
|  | | Parahippocampal gyrus | 0.002 | 0.06 | 0.98 | -0.001 | 0.005 | 0.86 |
|  | | Entorhinal cortex | 0.0003 | 0.06 | 1.00 | -0.0004 | 0.01 | 0.94 |
|  | | Amygdala | -0.05 | 0.06 | 0.40 | -0.003 | 0.01 | 0.63 |

Note. Results are derived from linear mixed effect models that examined interactions between plasma biomarkers and Aβ_42/40_ status on brain volume (adjusted for baseline age, sex, race, education level, estimated glomerular filtration rate (eGFR), total intracranial volume at age 70, and time-covariate interactions).

Cross-sectional effects reflect whether the association between each plasma biomarker and brain volume varies by Aβ_42/40_ status. Longitudinal effects reflect whether the association between each plasma biomarker and changes in brain volume vary by Aβ_42/40_ status. Cross-sectional effects reflect plasma biomarker × Aβ_42/40_ status interactions, whereas longitudinal effects reflect plasma biomarker × Aβ_42/40_ status × time interactions. *Remains significant after 0.05 FDR correction. *Abbreviations*: Aβ: amyloid-β; CSF: cerebral spinal fluid; GFAP: glial fibrillary acidic protein; NfL: neurofilament light chain; ROI: region of interest; pTau-181: tau phosphorylated at threonine-181; SE: standard error.

# Supplemental Table 8. Associations between baseline plasma biomarkers and cognitive performance

|  |  | Cross-sectional effects | | | Longitudinal effects | | |
| --- | --- | --- | --- | --- | --- | --- | --- |
| Biomarker | Cognitive domain | β | SE | *P* | β | SE | *P* |
| Aβ_42/40_ | Verbal Memory | -0.10 | 0.03 | 0.01 | 0.02 | 0.01 | <0.0001* |
|  | Attention | -0.005 | 0.03 | 0.87 | 0.01 | 0.004 | 0.16 |
|  | Executive function | -0.02 | 0.03 | 0.57 | 0.01 | 0.004 | 0.07 |
|  | Verbal Fluency | -0.02 | 0.03 | 0.63 | 0.01 | 0.003 | 0.08 |
|  | Visuospatial | -0.08 | 0.04 | 0.04 | 0.01 | 0.003 | 0.02* |
|  |  | β | SE | *P* | β | SE | *P* |
| pTau-181 | Verbal Memory | -0.001 | 0.04 | 0.97 | -0.02 | 0.01 | 0.01 |
|  | Attention | 0.03 | 0.03 | 0.28 | -0.01 | 0.004 | 0.05 |
|  | Executive function | -0.03 | 0.03 | 0.37 | -0.01 | 0.005 | 0.27 |
|  | Verbal Fluency | 0.04 | 0.04 | 0.25 | -0.01 | 0.004 | 0.03 |
|  | Visuospatial | -0.02 | 0.04 | 0.65 | -0.01 | 0.004 | 0.16 |
|  |  | β | SE | *P* | β | SE | *P* |
| GFAP | Verbal Memory | 0.02 | 0.04 | 0.55 | -0.01 | 0.01 | 0.08 |
|  | Attention | -0.003 | 0.03 | 0.92 | -0.0002 | 0.005 | 0.96 |
|  | Executive function | -0.01 | 0.03 | 0.80 | -0.01 | 0.005 | 0.26 |
|  | Verbal Fluency | 0.04 | 0.04 | 0.24 | -0.01 | 0.004 | 0.002* |
|  | Visuospatial | -0.04 | 0.04 | 0.35 | -0.004 | 0.004 | 0.34 |
|  |  | β | SE | *P* | β | SE | *P* |
| NfL | Verbal Memory | -0.01 | 0.04 | 0.80 | 0.004 | 0.006 | 0.48 |
|  | Attention | 0.04 | 0.03 | 0.20 | -0.002 | 0.004 | 0.66 |
|  | Executive function | 0.04 | 0.03 | 0.21 | -0.002 | 0.005 | 0.75 |
|  | Verbal Fluency | 0.07 | 0.04 | 0.04 | -0.01 | 0.004 | 0.12 |
|  | Visuospatial | -0.03 | 0.04 | 0.48 | 0.001 | 0.004 | 0.83 |

Note. Results are derived from linear mixed effect models adjusted for baseline age, sex, race, education level, estimated glomerular filtration rate (eGFR), and time-covariate interactions. Cross-sectional β values reflect the difference in cognitive performance per standard deviation (SD) increase in biomarker. Longitudinal β values reflect the annual change in cognitive performance per SD increase in biomarker. Cross-sectional effects reflect the main effect of each biomarker, whereas longitudinal effects reflect plasma biomarker × time interactions. *Remains significant after 0.05 FDR correction. *Abbreviations*: Aβ: amyloid-β; GFAP: glial fibrillary acidic protein; NfL: neurofilament light chain; pTau-181: tau phosphorylated at threonine-181; SE: standard error.

# Supplemental Table 9. Associations between baseline plasma biomarkers and cognitive performance (without eGFR as a covariate)

|  |  | Cross-sectional effects | | | Longitudinal effects | | |
| --- | --- | --- | --- | --- | --- | --- | --- |
| Biomarker | Cognitive domain | β | SE | *P* | β | SE | *P* |
| Aβ_42/40_ | Verbal Memory | -0.10 | 0.03 | 0.005* | 0.02 | 0.01 | <0.0001* |
|  | Attention | -0.005 | 0.03 | 0.87 | 0.01 | 0.004 | 0.19 |
|  | Executive function | -0.02 | 0.03 | 0.56 | 0.01 | 0.004 | 0.09 |
|  | Verbal Fluency | -0.02 | 0.03 | 0.63 | 0.01 | 0.003 | 0.10 |
|  | Visuospatial | -0.08 | 0.04 | 0.04 | 0.01 | 0.003 | 0.01* |
|  |  | β | SE | *P* | β | SE | *P* |
| pTau-181 | Verbal Memory | 0.01 | 0.04 | 0.71 | -0.01 | 0.01 | 0.01 |
|  | Attention | 0.04 | 0.03 | 0.26 | -0.01 | 0.004 | 0.07 |
|  | Executive function | -0.02 | 0.03 | 0.45 | -0.004 | 0.004 | 0.40 |
|  | Verbal Fluency | 0.04 | 0.04 | 0.26 | -0.01 | 0.004 | 0.07 |
|  | Visuospatial | -0.02 | 0.04 | 0.68 | -0.01 | 0.004 | 0.17 |
|  |  | β | SE | *P* | β | SE | *P* |
| GFAP | Verbal Memory | 0.03 | 0.04 | 0.43 | -0.01 | 0.01 | 0.07 |
|  | Attention | -0.002 | 0.03 | 0.94 | 0.0001 | 0.005 | 0.98 |
|  | Executive function | -0.01 | 0.03 | 0.84 | -0.005 | 0.005 | 0.31 |
|  | Verbal Fluency | 0.04 | 0.04 | 0.23 | -0.01 | 0.004 | 0.003* |
|  | Visuospatial | -0.04 | 0.04 | 0.38 | -0.004 | 0.004 | 0.31 |
|  |  | β | SE | *P* | β | SE | *P* |
| NfL | Verbal Memory | 0.01 | 0.04 | 0.77 | 0.002 | 0.01 | 0.70 |
|  | Attention | 0.04 | 0.03 | 0.18 | -0.001 | 0.004 | 0.74 |
|  | Executive function | 0.04 | 0.03 | 0.16 | -0.001 | 0.005 | 0.88 |
|  | Verbal Fluency | 0.07 | 0.03 | 0.04 | -0.01 | 0.004 | 0.16 |
|  | Visuospatial | -0.02 | 0.04 | 0.59 | 0.0002 | 0.004 | 0.97 |

Note. Results are derived from linear mixed effect models adjusted for baseline age, sex, race, education level, and time-covariate interactions. Cross-sectional β values reflect the difference in cognitive performance per standard deviation (SD) increase in biomarker. Longitudinal β values reflect the annual change in cognitive performance per SD increase in biomarker. Cross-sectional effects reflect the main effect of each plasma biomarker, whereas longitudinal effects reflect plasma biomarker × time interactions. *Remains significant after 0.05 FDR correction. *Abbreviations*: Aβ: amyloid-β; GFAP: glial fibrillary acidic protein; NfL: neurofilament light chain; pTau-181: tau phosphorylated at threonine-181; SE: standard error.

# Supplemental Table 10. Associations between baseline plasma biomarkers and cognitive performance (full model results)

*(See excel spreadsheet)*

# Supplemental Table 11. The effects of sex on the relationship between baseline plasma biomarkers and cognitive performance

|  |  | Cross-sectional effects | | | Longitudinal effects | | |
| --- | --- | --- | --- | --- | --- | --- | --- |
|  | Cognitive domain | β | SE | *P* | β | SE | *P* |
| Aβ_42/40_ × sex | Verbal Memory | -0.09 | 0.07 | 0.20 | 0.02 | 0.01 | 0.06 |
|  | Attention | -0.04 | 0.05 | 0.48 | 0.0001 | 0.01 | 0.99 |
|  | Executive function | 0.02 | 0.06 | 0.68 | -0.01 | 0.01 | 0.23 |
|  | Verbal Fluency | -0.05 | 0.06 | 0.42 | -0.002 | 0.01 | 0.83 |
|  | Visuospatial | 0.09 | 0.07 | 0.21 | -0.002 | 0.01 | 0.82 |
|  |  | β | SE | *P* | β | SE | *P* |
| pTau-181 × sex | Verbal Memory | -0.09 | 0.07 | 0.21 | 0.01 | 0.01 | 0.50 |
|  | Attention | 0.02 | 0.06 | 0.77 | -0.01 | 0.01 | 0.13 |
|  | Executive function | -0.09 | 0.06 | 0.15 | 0.004 | 0.01 | 0.64 |
|  | Verbal Fluency | 0.02 | 0.07 | 0.77 | 0.01 | 0.01 | 0.51 |
|  | Visuospatial | -0.05 | 0.08 | 0.50 | 0.01 | 0.01 | 0.09 |
|  |  | β | SE | *P* | β | SE | *P* |
| GFAP × sex | Verbal Memory | 0.08 | 0.07 | 0.23 | -0.01 | 0.01 | 0.18 |
|  | Attention | -0.01 | 0.05 | 0.92 | -0.01 | 0.01 | 0.41 |
|  | Executive function | -0.07 | 0.06 | 0.23 | -0.003 | 0.01 | 0.73 |
|  | Verbal Fluency | 0.02 | 0.06 | 0.78 | -0.004 | 0.01 | 0.55 |
|  | Visuospatial | 0.07 | 0.07 | 0.35 | -0.02 | 0.01 | 0.01 |
|  |  | β | SE | *P* | β | SE | *P* |
| NfL × sex | Verbal Memory | 0.003 | 0.06 | 0.96 | 0.001 | 0.01 | 0.93 |
|  | Attention | 0.04 | 0.05 | 0.46 | -0.001 | 0.01 | 0.91 |
|  | Executive function | -0.05 | 0.05 | 0.36 | 0.01 | 0.01 | 0.37 |
|  | Verbal Fluency | 0.04 | 0.06 | 0.54 | -0.003 | 0.01 | 0.63 |
|  | Visuospatial | 0.10 | 0.07 | 0.15 | -0.01 | 0.01 | 0.22 |

Note. Results are derived from linear mixed effect models that examined interactions between plasma biomarkers and sex on cognitive performance (adjusted for baseline age, sex, race, education level, estimated glomerular filtration rate (eGFR), and time-covariate interactions). Cross-sectional effects reflect whether the association between each plasma biomarker and cognitive performance varies by sex. Longitudinal effects reflect whether the association between each biomarker and changes in cognitive performance vary by sex. Cross-sectional effects reflect plasma biomarker × sex interactions, whereas longitudinal effects reflect plasma biomarker × sex × time interactions. No associations survived FDR correction. *Abbreviations*: Aβ: amyloid-β; GFAP: glial fibrillary acidic protein; NfL: neurofilament light chain; pTau-181: tau phosphorylated at threonine-181; SE: standard error.

# Supplemental Table 12. Modifier-specific (sex and Aβ_42/40_ status) estimates of the relationship between baseline plasma biomarkers and change in cognitive performance

| Significant 3-way interaction |  | Modifier-specific estimates | | | | | |
| --- | --- | --- | --- | --- | --- | --- | --- |
|  |  | Male | | | Female | | |
|  |  | β | SE | *P* | β | SE | *P* |
| GFAP × sex × time | Visuospatial | -0.02 | 0.01 | 0.01 | 0.004 | 0.01 | 0.49 |
|  |  | Low Aβ_42/40_ | | | High Aβ_42/40_ | | |
|  |  | β | SE | *P* | β | SE | *P* |
| GFAP × Aβ_42/40_ status × time | Verbal memory | -0.02 | 0.01 | 0.02 | 0.01 | 0.01 | 0.38 |
|  |  | Low Aβ_42/40_ | | | High Aβ_42/40_ | | |
|  |  | β | SE | *P* | β | SE | *P* |
| pTau-181 × Aβ_42/40_ status × time | Verbal memory | -0.03 | 0.01 | 0.004 | -0.001 | 0.01 | 0.88 |

Note. β values reflect modifier-specific estimates that are derived from the linear mixed effect models that examined interactions between plasma biomarkers and sex, and Aβ_42/40_ status on cognitive performance (adjusted for baseline age, sex, race, education level, estimated glomerular filtration rate (eGFR), total intracranial volume at age 70, and time-covariate interactions). Only longitudinal effects are presented in the table. Longitudinal effects reflect whether the association between each plasma biomarker and changes in cognitive performance vary by sex, or Aβ_42/40_ status. Only results from significant three-way interactions are presented. Multiple comparison correction was not completed these analyses.

# Supplemental Table 13. The effects of *APOE*ε4 status on the relationship between baseline plasma biomarkers and cognitive performance

|  |  | Cross-sectional effects | | | Longitudinal effects | | |
| --- | --- | --- | --- | --- | --- | --- | --- |
|  | Cognitive domain | β | SE | *P* | β | SE | *P* |
| Aβ_42/40_ × *APOE*ε4 | Verbal Memory | 0.13 | 0.08 | 0.12 | -0.01 | 0.01 | 0.47 |
|  | Attention | 0.15 | 0.07 | 0.02 | -0.01 | 0.01 | 0.20 |
|  | Executive function | 0.08 | 0.07 | 0.24 | -0.01 | 0.01 | 0.18 |
|  | Verbal Fluency | 0.02 | 0.07 | 0.78 | -0.001 | 0.01 | 0.94 |
|  | Visuospatial | 0.03 | 0.09 | 0.69 | 0.001 | 0.01 | 0.94 |
|  |  | β | SE | *P* | β | SE | *P* |
| pTau-181 × *APOE*ε4 | Verbal Memory | 0.09 | 0.08 | 0.27 | -0.03 | 0.01 | 0.06 |
|  | Attention | 0.01 | 0.07 | 0.93 | 0.01 | 0.01 | 0.43 |
|  | Executive function | 0.02 | 0.07 | 0.76 | 0.01 | 0.01 | 0.37 |
|  | Verbal Fluency | 0.10 | 0.08 | 0.20 | -0.01 | 0.01 | 0.23 |
|  | Visuospatial | 0.03 | 0.09 | 0.72 | -0.004 | 0.01 | 0.70 |
|  |  | β | SE | *P* | β | SE | *P* |
| GFAP × *APOE*ε4 | Verbal Memory | 0.03 | 0.08 | 0.68 | -0.002 | 0.01 | 0.85 |
|  | Attention | -0.12 | 0.06 | 0.05 | 0.01 | 0.01 | 0.10 |
|  | Executive function | -0.02 | 0.06 | 0.74 | -0.002 | 0.01 | 0.83 |
|  | Verbal Fluency | 0.003 | 0.07 | 0.97 | -0.004 | 0.01 | 0.65 |
|  | Visuospatial | 0.10 | 0.08 | 0.21 | 0.003 | 0.01 | 0.70 |
|  |  | β | SE | *P* | β | SE | *P* |
| NfL × *APOE*ε4 | Verbal Memory | 0.09 | 0.08 | 0.25 | -0.01 | 0.01 | 0.35 |
|  | Attention | -0.18 | 0.07 | 0.01 | 0.01 | 0.01 | 0.12 |
|  | Executive function | -0.01 | 0.07 | 0.83 | -0.01 | 0.01 | 0.25 |
|  | Verbal Fluency | -0.003 | 0.07 | 0.96 | -0.01 | 0.01 | 0.12 |
|  | Visuospatial | -0.001 | 0.09 | 0.99 | 0.01 | 0.01 | 0.26 |

Note. Results are derived from linear mixed effect models that examined interactions between plasma biomarkers and *APOE*ε4 status on cognitive performance (adjusted for baseline age, sex, race, education level, estimated glomerular filtration rate (eGFR), and time-covariate interactions). Cross-sectional effects reflect whether the association between each plasma biomarker and cognitive performance varies by *APOE*ε4 status. Longitudinal effects reflect whether the association between each biomarker and changes in cognitive performance vary by *APOE*ε4 status. Cross-sectional effects reflect plasma biomarker × *APOE*ε4 status interactions, whereas longitudinal effects reflect plasma biomarker × *APOE*ε4 status × time interactions. No associations survived FDR correction.

*Abbreviations*: Aβ: amyloid-β; GFAP: glial fibrillary acidic protein; NfL: neurofilament light chain; pTau-181: tau phosphorylated at threonine-181; SE: standard error.

# Supplemental Table 14. The effects of Aβ_42/40_ status on the relationship between baseline plasma biomarkers and cognitive performance

|  |  | Cross-sectional effects | | | Longitudinal effects | | |
| --- | --- | --- | --- | --- | --- | --- | --- |
|  | Cognitive domain | β | SE | *P* | β | SE | *P* |
| pTau-181 × Aβ_42/40_ status | Verbal Memory | 0.02 | 0.07 | 0.79 | -0.02 | 0.01 | 0.03 |
|  | Attention | 0.04 | 0.06 | 0.55 | -0.01 | 0.01 | 0.08 |
|  | Executive function | 0.03 | 0.06 | 0.66 | -0.001 | 0.01 | 0.95 |
|  | Verbal Fluency | 0.003 | 0.07 | 0.97 | -0.01 | 0.01 | 0.11 |
|  | Visuospatial | -0.04 | 0.08 | 0.59 | -0.01 | 0.01 | 0.43 |
|  |  | β | SE | *P* | β | SE | *P* |
| GFAP × Aβ_42/40_ status | Verbal Memory | 0.001 | 0.07 | 0.99 | -0.03 | 0.01 | 0.02 |
|  | Attention | -0.02 | 0.06 | 0.70 | -0.01 | 0.01 | 0.12 |
|  | Executive function | -0.05 | 0.06 | 0.44 | -0.01 | 0.01 | 0.12 |
|  | Verbal Fluency | -0.05 | 0.07 | 0.42 | -0.01 | 0.01 | 0.14 |
|  | Visuospatial | 0.03 | 0.08 | 0.71 | -0.01 | 0.01 | 0.07 |
|  |  | β | SE | *P* | β | SE | *P* |
| NfL × Aβ_42/40_ status | Verbal Memory | -0.03 | 0.07 | 0.63 | -0.01 | 0.01 | 0.61 |
|  | Attention | 0.04 | 0.05 | 0.45 | -0.01 | 0.01 | 0.38 |
|  | Executive function | -0.05 | 0.06 | 0.38 | 0.005 | 0.01 | 0.56 |
|  | Verbal Fluency | 0.02 | 0.06 | 0.77 | -0.003 | 0.01 | 0.66 |
|  | Visuospatial | 0.01 | 0.07 | 0.86 | -0.005 | 0.01 | 0.52 |

Note. Results are derived from linear mixed effect models that examined interactions between plasma biomarkers and Aβ_42/40_ status on cognitive performance (adjusted for baseline age, sex, race, education level, estimated glomerular filtration rate (eGFR), and time-covariate interactions). Cross-sectional effects reflect whether the association between each plasma biomarker and cognitive performance varies by Aβ_42/40_ status. Longitudinal effects reflect whether the association between each biomarker and changes in cognitive performance vary by Aβ_42/40_ status. Cross-sectional effects reflect plasma biomarker × Aβ_42/40_ status interactions, whereas longitudinal effects reflect plasma biomarker × Aβ_42/40_ status × time interactions. No associations survived FDR correction. *Abbreviations*: Aβ: amyloid-β; GFAP: glial fibrillary acidic protein; NfL: neurofilament light chain; pTau-181: tau phosphorylated at threonine-181; SE: standard error.

# Supplemental Figure 1. Effect of baseline plasma biomarkers on age-related trajectories of brain volume and verbal memory, stratified by Aβ_42/40_ status

a) Results are derived from linear mixed effect models (models were adjusted for baseline age, sex, race, education level, eGFR, total intracranial volume at age 70, and the interaction between covariates and time) that examined Aβ_42/40_ as a moderator of the association between plasma biomarkers and longitudinal change in brain volume. Results show the effects of pTau-181 on age-related longitudinal trajectories in ventricular and hippocampal volume by amyloid status. b) Results are derived from linear mixed effect models (models were adjusted for baseline age, sex, race, education level, eGFR, and the interaction between covariates and time) that examined Aβ_42/40_ as a moderator of the association between plasma biomarkers and longitudinal change in cognitive performance. Results show the effects of pTau-181 and GFAP on age-related longitudinal trajectories in verbal memory by Aβ_42/40_ status. *Abbreviations*: Aβ: amyloid-β; GFAP: glial fibrillary acidic protein; pTau-181: tau phosphorylated at threonine-181; SD: standard deviation.
